# 

**Published:** 1893-04

**Authors:** 


### CONTENTS FOR APRIL, 1893.

PAGE.

University of Buffalo ..... 517

ORIGINAL COMMUNICATIONS.

The Diagnosis of Gastric Disorders. By ALLEN A. JONES, M. D. ..... 535
Intestinal Irrigation—Enteroclysis. By FREDERIC W. BARTLETT, M. D. ..... 542

CLINICAL REPORTS.

Report of a Case of Acute Bilateral Psoitis ..... 547
Laceration of Cervix, Endometritis, Lateral Displacement of Uterus, Pelvic Peritonitis Following Abortion ..... 549
Report of a Case of Pressure Neuritis of Both Sciatic Nerves ..... 552

PROGRESS IN MEDICAL SCIENCE.

Chemistry ..... 554
Surgery ..... 556
Abstracts ..... 559

EDITORIAL.

The New National Quarantine Law ..... 560
Topics of the Month ..... 562
Personal ..... 564
Obituary ..... 564
Society Meetings ..... 565
Academy of Medicine Notes ..... 570
College Notes ..... 571
Book Reviews ..... 571
Miscellany ..... 578

